# Optimal orientation in flows: providing a benchmark for animal movement strategies

**DOI:** 10.1098/rsif.2014.0588

**Published:** 2014-10-06

**Authors:** James D. McLaren, Judy Shamoun-Baranes, Adriaan M. Dokter, Raymond H. G. Klaassen, Willem Bouten

**Affiliations:** 1 Computational Geo-Ecology, Institute for Biodiversity and Ecosystem Dynamics, University of Amsterdam, Amsterdam, The Netherlands; 2 Dutch Centre for Avian Migration and Demography, Department of Animal Ecology, Netherlands Institute for Ecology (NIOO-KNAW), Wageningen, The Netherlands; 3 Dutch Montagu's Harrier Foundation, Animal Ecology Group, University of Groningen, Groningen, The Netherlands

**Keywords:** flow orientation, animal navigation, migration, lateral drift, optimization, movement ecology

## Abstract

Animal movements in air and water can be strongly affected by experienced flow. While various flow-orientation strategies have been proposed and observed, their performance in variable flow conditions remains unclear. We apply control theory to establish a benchmark for time-minimizing (optimal) orientation. We then define optimal orientation for movement in steady flow patterns and, using dynamic wind data, for short-distance mass movements of thrushes (*Turdus* sp.) and 6000 km non-stop migratory flights by great snipes, *Gallinago media.* Relative to the optimal benchmark, we assess the efficiency (travel speed) and reliability (success rate) of three generic orientation strategies: full compensation for lateral drift, vector orientation (single-heading movement) and goal orientation (continually heading towards the goal). Optimal orientation is characterized by detours to regions of high flow support, especially when flow speeds approach and exceed the animal's self-propelled speed. In strong predictable flow (short distance thrush flights), vector orientation adjusted to flow on departure is nearly optimal, whereas for unpredictable flow (inter-continental snipe flights), only goal orientation was near-optimally reliable and efficient. Optimal orientation provides a benchmark for assessing efficiency of responses to complex flow conditions, thereby offering insight into adaptive flow-orientation across taxa in the light of flow strength, predictability and navigation capacity.

## 1. Introduction

Advection by the surrounding flow can be of paramount importance to an animal moving in water or air [1–3]. By adjusting its body orientation (heading) or self-propelled speed relative to the moving flow (self-speed), an animal can influence its resultant track direction and speed of travel in relation to the ground (ground speed; see Glossary for terms relevant to this study) [3,4]. Hence an organism's response to flow conditions, or lack thereof, will influence its travel duration, route, total energy expenditure, and whether a destination (goal) can actually be reached [5,6]. Not surprisingly, animals across taxa have evolved mechanisms to gauge and react to the surrounding flow [2,7–10].

In nature, flow conditions often vary unpredictably, especially at longer spatial and temporal scales [11]. This can present a formidable challenge to an animal aiming to minimize its duration of travel to a specific goal [12]. Successful arrival requires adjustment of headings to compensate for any cumulative lateral drift. This can be accomplished either by gauging and compensating for currently experienced drift or by using a map sense to reorient towards the goal [12]. The time-minimizing orientation strategy to reach a goal in unpredictable flow has been proposed to involve minimizing the remaining distance to the goal in a sequence of steps, resulting in an increased degree of compensation on approach to the goal [12,13]. However, in order to determine how reliable and efficient different flow strategies are in given flow conditions, it is helpful to have a benchmark for the absolute fastest, i.e. time-minimizing response. This response (hereafter, optimal orientation) assumes that an animal has perfect information about flow conditions at any time and location, analogous to the omniscient forager in optimal foraging theory [14].

Our primary aim is to quantify optimal orientation to specified goals assuming constant self-speeds, as a benchmark for studying the efficiency of animal movement in horizontal flow regimes. While ignoring vertical structure is inappropriate for soaring and buoyant taxa [15–17], long-distance movements among some flapping [18–20] and swimming [21,22] taxa seem to be largely horizontal once selection of appropriate vertical strata is made (but see e.g. [23,24]). For simplicity, we also assume constant self-speeds and purely horizontal movement. We solve optimal orientation using results from optimal control theory, which reduces the seemingly incalculable problem of solving optimal headings at every potential point in space and time to an initial-value problem in which only the initial heading needs to be solved [25]. The origins of optimal control theory can be traced from Bernoulli in the seventeenth century [26] to twentieth century aviation and pursuit studies [27–29]. Somewhat counterintuitively, time-optimal orientation in horizontal flow involves continual alteration of headings to steer towards flow which is less supportive of the current travel direction [27,28,30]. Though seldom applied in ecology, optimal control was recently used to estimate minimum wind speeds required for albatrosses to maintain dynamic soaring in vertical wind shear [31].

Over larger distances, we expect that animals will not be capable of achieving or perhaps even approaching the omniscience required to optimally orient. Moreover, the ability to gauge or predict flow (e.g. [32,33]) remains challenging to assess, as does the accuracy and hierarchy among navigational mechanisms, i.e. when and to what extent these are used during movement [34,35]. Nonetheless by comparing the extent to which other strategies approach the time-efficiency of optimal orientation, we can gain insight into the adaptive benefit of flow information and more sophisticated orientation strategies. Our second aim is therefore to demonstrate how optimal orientation can be used as a benchmark to assess the absolute and relative efficacy of proposed animal orientation strategies. We evaluate the robustness to flow variability of three generic flow-orientation strategies, assessed by reliability (proportional arrival over varying conditions, hereafter success rate, *p*_A_) and efficiency (travel speed relative to optimal orientation, *ɛ*):

(1) Full compensation: based on continual adjustment of heading to maintain a constant track direction (great circles or rhumblines on a sphere).
(2) Vector orientation: based on a single heading, set on departure and ignoring drift thereafter (arrival is possible to the extent that the heading can be adjusted to compensate for any cumulative lateral drift, see [36,37]).
(3) Goal orientation: based on continually heading towards the goal using a map sense, i.e. ignoring instantaneous lateral drift.

These strategies and their relevance to animal movement are described in more detail in appendix A (see also Glossary and [3,4]). They are by no means exhaustive, necessarily attainable or expected to be favourable in given flow scenarios. While more sophisticated responses to horizontal flow have been proposed [12,38], underlying animal orientation strategies remain to be quantified.

We first determined optimal orientation and assessed each generic orientation strategy in two commonly occurring flow patterns: (i) a gradient in lateral flow along the journey (hereafter, shear flow), emulating, for example, the transition between trade winds and westerly winds with increasing latitude for a migratory bird [2] and (ii) purely rotational flow, emulating mid-latitude (anti-)cyclones [39]. For each strategy, we determined the resulting flow support (proportional gain in travel speed due to flow) for movement within each pattern with various flow strengths, and assessed the resultant reliability and efficiency. For simplicity, we assumed steady (time-invariant) flow.

In addition to these steady flow patterns, we also determined optimal orientation and assessed the generic strategies in time-varying flow for two avian migration systems using an individual-based model [36] together with publically available global wind data [40,41]. First, we simulated 14 mass nocturnal migration events across the North Sea which included large numbers of Eurasian redwings (*Turdus iliacus*) and song thrushes (*Turdus philomelos*) [42] of Scandinavian origin. Analysis of radar tracking revealed these events involved a flexible reaction to wind facilitating arrival in The Netherlands [42], which according to ring-recovery data is a preferred autumn destination for these thrush populations [43,44]. In reconstructing these events, we therefore assumed that these migrants aimed to arrive on land within 100 km of a specific goal location in The Netherlands (53° N 6° W), and that flow was predictable to the extent that vector-orienting migrants adjusted their headings on departure to ensure arrival. Secondly, we simulated 33 seasons of long and fast non-stop flights by great snipes (*Gallinago media*) from Scandinavia to within 250 km of a location in a prevalent stopover area in West Africa ([45] and RHG Klaassen 2011-2012, unpublished data). We assumed that great snipes could not predict flow conditions over continental distances, so we chose a single vector-orienting heading for the entire 33-year period which maximized the resultant overall success rate (cf. [37]).

In summary, we quantify optimal orientation in horizontal flow for a time-minimizing animal travelling to a specific destination, providing a benchmark to evaluate different possible orientation strategies, and demonstrate its use in evaluating generic orientation strategies in steady flow patterns and contrasting migration systems in time-varying flow.

## 2. Material and methods

### 2.1. Orientation in steady flow patterns

An animal's trajectory on a horizontal *x*–*y* plane over time (*t*) can be determined via its velocity components relative to the ground, *u*(*t*) and *v*(*t*), respectively. Each velocity component is a vector sum of the flow velocity and the animal's self-propelled (self-speed) velocity components:2.1
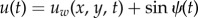
2.2
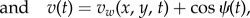
where *u_w_* and *v_w_* are the *x* and *y* flow velocity components, respectively, and *ψ* = *ψ*(*t*) is the migrant's heading clockwise from the *y*-axis. Equations (2.1) and (2.2) are scaled to the self-speed *V_a_*, i.e. spatially to a scale of flow, *L* and to the travel time in the absence of flow, *L*/*V_a_*. In this scaling, the initial goal distance *D* is also the travel duration in the absence of flow. Using calculus of variations or optimal control theory, it can be shown that optimal, i.e. time-minimizing, headings follow the classic Zermelo solution [27]:2.3
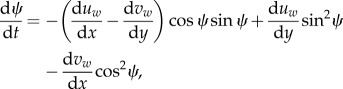
(in [27,30], headings are defined anticlockwise from the *x*-axis, resulting in the right-hand side of equation (2.3) being of opposite sign). From equation (2.3), it can be shown that once initial headings are chosen, optimally orienting migrants steer continually away from whichever side has higher flow support relative to the current travel direction [28,30]. Note that in uniform flow, the right-hand side of equation (2.3) is zero, demonstrating (cf. [13]) that full compensation is in this case time-optimal (being the only way to arrive at the goal with a constant heading).

Shear flow is characterized by a gradient in lateral flow along the initial goal direction2.4
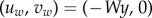


and rotational flow by radially increasing flow speed (as modelled for cyclones and anticyclones, see [39])2.5
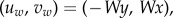
where *W* is the flow strength (maximum flow speed relative to the animal's self-speed). We scaled the shear flow case to the initial goal distance and the radius of flow in the rotational case, i.e. the dimensionless initial goal distance is *D* = 1 for shear flow and *D* = 2 for movement through rotational flow. Since the initial goal direction is along the *y*-axis, the initial and goal locations are (*x*_0_, *y*_0_) = (0, 0) and (*x_f_*, *y_f_*) = (0, 1) for movement through shear flow and (*x*_0_, *y*_0_) = (0, −1) and (*x_f_*, *y_f_*) = (0, 1) through rotational flow. For these configurations, analytical formulae for trajectories were derived for vector orientation (*x^c^*(*t*), *y^c^*(*t*)) and optimal orientation (x*(*t*), y*(*t*)) via eqn 32 in [30]. This facilitated solving initial headings 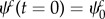 and 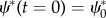 with vector and optimal orientation, respectively. For each candidate's initial heading, the closest approach to the goal was determined using MATLAB's minimizing routine fminbnd. Vector-orienting and optimal trajectories in shear flow are
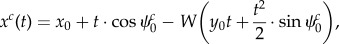

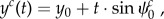

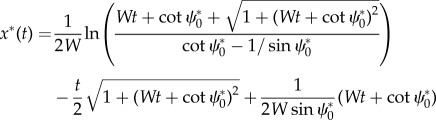

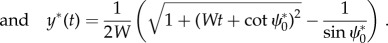


For the rotational case, vector-orienting trajectories (*x^c^*(*t*), *y^c^*(*t*)) are readily solved analytically
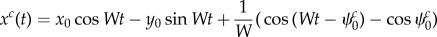

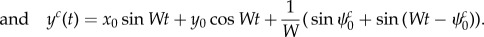
Optimal headings change linearly in time in rotational flow 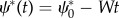, and optimal trajectories follow (via eqn 30 in [30]):
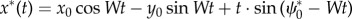

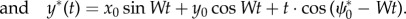


For both patterns, trajectories with goal orientation (*x*^G^(*t*), *y*^G^(*t*)) and full compensation (*x*^F^(*t*), *y*^F^(*t*)) were computed numerically using equations (2.1)–(2.2) and (2.4)–(2.5) with dimensionless time steps of 10^−5^, and according to the current heading. Goal-oriented headings *ψ*^G^(*t*) were updated to the current goal direction
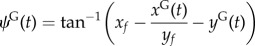
and headings during full compensation *ψ*^F^(*t*) were updated to counteract the current lateral flow
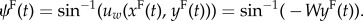


Travel durations for vector-orienting *T^c^* and fully compensating *T*^F^ individuals could be determined analytically as a function of the maximum flow speed *W*. In shear flow, these are
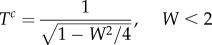

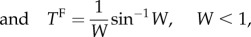
and for travel directly through rotational flow
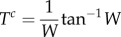

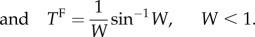


We also simulated movement wholly contained within rotational flow, varying the departure location at regularly spaced intervals within one quadrant (shaded region in figure 3*a,b*, Δ*x*_0_ = Δ*y*_0_ = 0.01, *N* = 12 959), and setting the initial goal distance to one radius distant on the opposite side of the flow pattern (i.e. (*x_f_*, *y_f_*) = (*x*_0_, *y*_0_ − 1)). For these locations, initial headings and trajectories were solved numerically for vector and optimal orientation, as were trajectories for goal orientation and full compensation.

### 2.2. Simulating avian migration in time-varying winds

To simulate long-distance migration, we resolved motion on a spherical surface, i.e. the rates of change in longitude Ø = Ø(*t*) and latitude *θ* = *θ* (*t*):
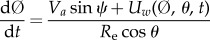

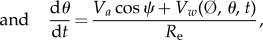
where *U_w_* and *V_w_* are the (dimensional) eastward and northward flow velocities, *R*_e_ is the Earth's radius, and the heading *ψ* is measured clockwise from geographic north. Zermelo's solution on the sphere becomes2.6
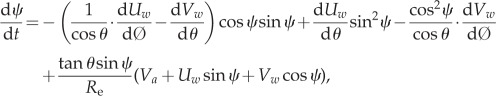
where the last term accounts for the Earth's curvature [25]. Here, we have neglected the vertical motion required to maintain altitude over the spherical Earth [28]. Finally, we note that with all strategies, the Coriolis effect [46] is assumed to be either insignificant or adjusted for by the animal in question.

To simulate optimal orientation in non-stop flight in time-varying winds, we modified the individual-based model in [36] to solve equation (2.6). Initial and goal locations, departure dates, times and flight characteristics were chosen to match each migration system. Departures in all simulations took place at 1 h following civil dusk, and we further assumed sufficient fuel loads to reach goal destinations. Non-stop flights of *Turdus* thrushes across the North Sea were simulated using wind data from the NCEP-NCAR reanalysis dataset [40] for 14 known mass migration events September–November 2006–2008 between Norway and The Netherlands [42]. We assumed departures from inland Norway (60° N 8.5° E) to within 100 km of a 794 km distant goal in The Netherlands (53° N 6° E), flight at 925 mb pressure level (*ca* 800 m above mean sea level (AMSL)) and self-speeds of 12 m s^−1^, appropriate for *Turdus* thrushes [47]. Simulated great snipes departed 16–30 August 1979–2011 from a location in Scandinavia (63° N, 12° E) to within 250 km of a 5909 km distant goal (10° N, 8° E) in a prevalent stopover region in West Africa, as evidenced by great snipes tracked using geolocation ([45] and RHG Klaassen 2011-2012, unpublished data). Based on these data, we assumed constant self-speeds (20 m s^−1^) and flight at 700 mb (*ca* 3000 m AMSL). To ensure unbiased comparisons between strategies, including unsuccessful dates, differences in efficiency between strategies were assessed using Wilcoxon's non-parametric two-way signed rank test.

Headings for full compensation and goal orientation were obtained in relation to orthodromic (great circle) directions at each time step [48]. Since optimal orientation theoretically involves altering headings according to exact spatial derivatives of flow (equation (2.6)) we linearly interpolated the wind data spatially and temporally at each time step. Solutions can furthermore be very sensitive to small errors in calculated headings (e.g. [25,49]), so we used time steps of 2 min for thrush simulations and 1 min for snipe simulations. While these resolutions go far beyond that of the original wind data (*ca* 1° and 1 h, [40]), they enabled precise calculations of benchmarks and accurate assessment of the generic strategies. To avoid convergence to local minima, initial headings were therefore determined within small intervals for each departure date (1° for thrush simulations and 0.05° for great snipe simulations) using a standard search routine (MATLAB's fminbnd) and the overall time-minimal initial heading chosen among all intervals. For 25 of the 495 simulated great snipe flights, even smaller intervals were required; these were resolved iteratively by visually comparing time-minimizing trajectories in successively smaller intervals until convergence was achieved.

## 3. Results

### 3.1. Movement in steady flow patterns

**Figure 1.**
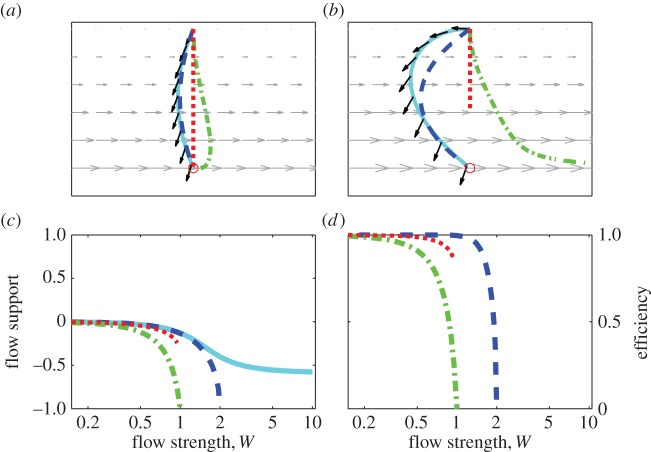
Orientation in shear flow. For movement through shear flow, trajectories to goals (marked with an O) with optimal orientation (cyan lines with black arrows representing optimal headings), vector orientation (dashed blue lines), goal orientation (dot-dashed green lines) and full compensation (dotted red lines) in moderately flow, *W* = 0.8, (*a*) and strong flow, *W* = 1.7, (*b*). (*c*) Flow support (proportional gain in travel speed) and efficiency (travel speed relative to optimal speed) in relation to flow strength *W* for each strategy. Note that all results are independent of spatial scale and that the *y*-axis points downwards.

**Figure 2.**
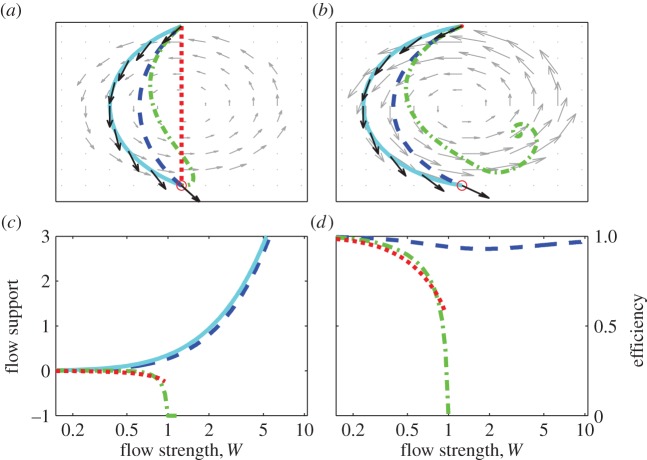
Orientation through rotational flow. For movement through rotational flow, trajectories in moderate (*a*) and strong flow (*b*), and flow support (*c*) and efficiency (*d*) as a function of flow strength. Details otherwise as in figure 1.

**Figure 3.**
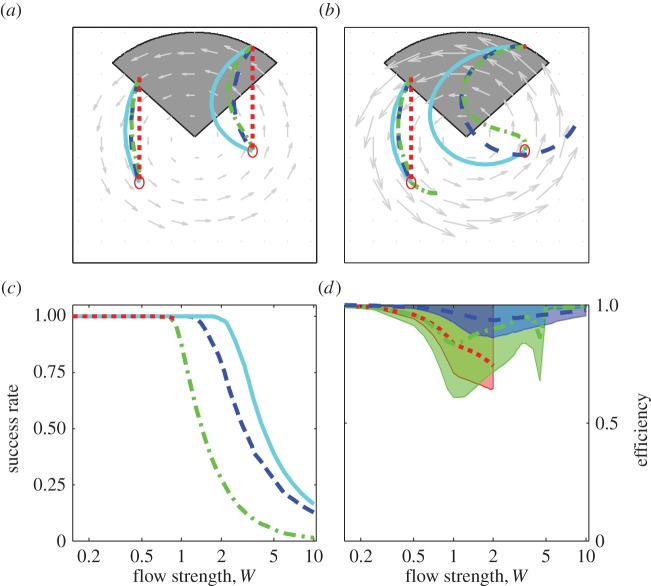
Orientation within rotational flow. For movement from different departure locations within rotational flow (shaded grey areas in (*a,b*)), trajectories in moderate (*a*) and strong flow (*b*) and for each strategy, success rates (*c*) and median efficiency among departure locations (*d*), with shaded area representing quartile range. Details otherwise as in figure 1.

We first present results of simulated movement through shear and rotational flow patterns and then, to account for variability in potential flow support en route, of movements from various departure locations within rotational flow patterns. For all simulations, the flow strength *W* (maximal flow speed relative to self-speed) was varied between 0 and 10, i.e. up to about twice the range encountered among taxa in fluid media [3]. Trajectories are shown for movement in flows of moderate strength (*W* = 0.8) and flows exceeding self-speeds (*W* = 1.7), spanning typical maximal values encountered by birds and by full-grown fish and turtles (cf. fig. 2 in [3]). Figures 1–3 graphically summarize results for each strategy for movement through shear (figure 1) and rotational flow patterns (figure 2) and from various departure locations within rotational flow patterns (figure 3).

Trajectories through weak to moderate flow (e.g. *W* = 0.8, figure 1*a*) differ much less between strategies than in strong flow (e.g. *W* = 1.7, figure 1*b*). Optimal orientation (cyan lines) takes advantage of the strong flow near the goal by effectively over-compensating for the initially weak lateral drift, resulting in upstream travel, and altering headings (depicted by black arrows in figure 1*a,b*) to reduce compensation on approach to the goal. The resultant detour contrasts with both the straight path resulting from full compensation (dotted red lines) and the initial downstream drift resulting from goal orientation (dot-dashed green lines). Flow-adjusted vector orientation (dashed blue lines) resembles optimal orientation in over-compensating on departure and initial upstream travel. Movement in pure shear flow remains unsupportive regardless of strategy, as illustrated by the increasingly negative flow support (proportional gain in travel speed due to flow) with increasing flow strength (figure 1*c*). This is also evident from the efficiency of each generic strategy (figure 1*d*), being nearly optimally efficient (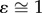) in weak flow (*W* < 0.5), but decreasing rapidly to zero in moderate to strong flow. Arrival with full compensation or goal orientation becomes infeasible at the point where *W* = 1, since individuals fully compensating for a purely lateral flow of equal strength become stationary. Vector orientation remains feasible as long as *W* < 2 since the mean lateral flow strength is *W*/2.

With movement through rotational flow, trajectories differed between strategies in both moderately strong flow (e.g. *W* = 0.8, figure 2*a*) and in strong flow (e.g. *W* = 1.7, figure 2*b*). Here, trajectories with both optimal orientation and vector orientation follow the counter-clockwise rotation of the flow, drifting far from the direct route with full compensation (figure 2*a*,*b*). Optimal orientation involves over-drift, i.e. heading partly towards the lateral flow, whereas vector orientation is equivalent to full drift in this particular example since flow is balanced en route. In strong flow (figure 2*b*), a fully compensating animal cannot move forward on departure, and a goal-orienting animal winds up spiralling inwards towards a stationary point remote from the goal. This is reflected in the dependence of flow support on flow strength (figure 2*c*): flow support with vector orientation and optimal orientation is always positive and increases with increasing flow strength, but is always negative with full compensation and goal orientation. Vector orientation, by taking advantage of the inherent balance in rotational flow patterns, remains feasible and efficient (*ɛ* > 0.9) regardless of flow strength (figure 2*d*).

For all strategies, varying the departure location in rotational flow (the shaded region in figure 3*a*,*b*) affects both the feasibility and efficiency of travel within the flow pattern. Sample trajectories are shown in moderate (*W* = 0.8, figure 3*a*) and strong flow (*W* = 1.7, figure 3*b*). Trajectories in supportive flow (e.g. the left-hand trajectories in figure 3*a*,*b*) differ much less with flow strength or between strategies compared to trajectories in opposing flow (e.g. right-hand trajectories, with goal orientation the only feasible generic strategy for *W* = 1.7). Figure 3*c* depicts success rates over all departure locations, showing that arrival is not always possible in very strong flow (*W* > 2, at least without first exiting the rotational system). In strong flow, optimal orientation always has the highest success rate, followed by vector orientation, then goal orientation, whereas full compensation is infeasible. Efficiencies for each strategy (figure 3*d*) further indicate that vector orientation is the most reliable and efficient among the tested generic strategies. The apparent increase in efficiency with very strong flow (*W* > 1) reflects that only highly supportive routes remain feasible.

### 3.2. Migration simulations

**Figure 4.**
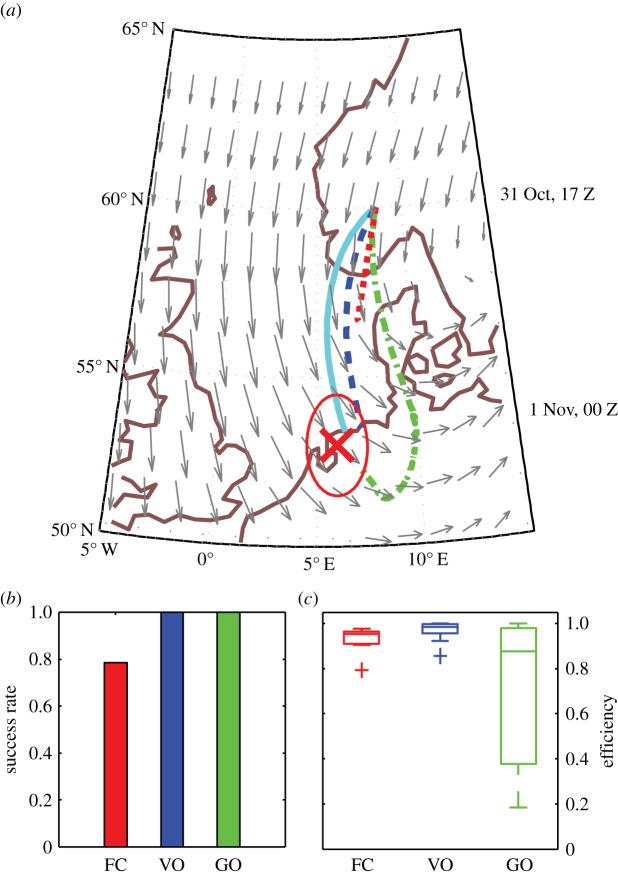
Simulated songbird migration across the North Sea. Trajectories of simulated *Turdus* thrushes (*a*) departing Norway on 31 October 2006 to land within a 100 km radius (outlined in red) of a goal located in The Netherlands (red cross), and considering 14 mass North Sea crossing events (September–November 2006–2007), success rates (*b*) and boxplots of efficiency (*c*) for each strategy: optimal orientation (cyan lines), vector orientation (VO, dashed blue lines), goal orientation (GO, dot-dashed green lines) and full compensation (FC, dotted red lines). Wind quivers (grey arrows) depicting wind speed and direction are scaled to 26 m s^−1^ and synchronized with optimally orienting migrants at the same latitude (see time stamps on right of maps).

Results for simulated North Sea crossings by thrushes are summarized in figure 4, with trajectories for each strategy in strong winds on 31 October 2006 (*W* = 2.16, figure 4*a*) and, considering all 14 mass migration events, success rate (figure 4*b*) and boxplots of efficiency (figure 4*c*). Trajectories are reminiscent of movement in strong rotational flow (figure 2*b*): simulated flight durations (and efficiencies) for this date were 8.7 h (1.0) with optimal orientation, 9.0 h (0.97) with vector orientation, 39 h (0.22) with goal orientation and with fully compensating birds failing to arrive (simulations were terminated when wind speeds exceeded airspeeds). Considering all 14 events, simulated flights were always successful (*p*_A_ = 1.0) except with full compensation which was unsuccessful on three of 14 nights (*p*_A_ = 0.79). Vector orientation was the most efficient strategy (median efficiency 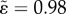 versus 0.95 with full compensation and 0.88 with goal orientation, Wilcoxon's signed rank = 4, *p* < 0.001). This was even more apparent on simulations of the six nights where mean wind speeds exceeded the modelled self-speed, with full compensation resulting in successful arrival on only three nights, and poor efficiency with goal orientation 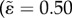 versus 0.95 and 0.96 with full compensation and vector orientation, respectively).

**Figure 5.**
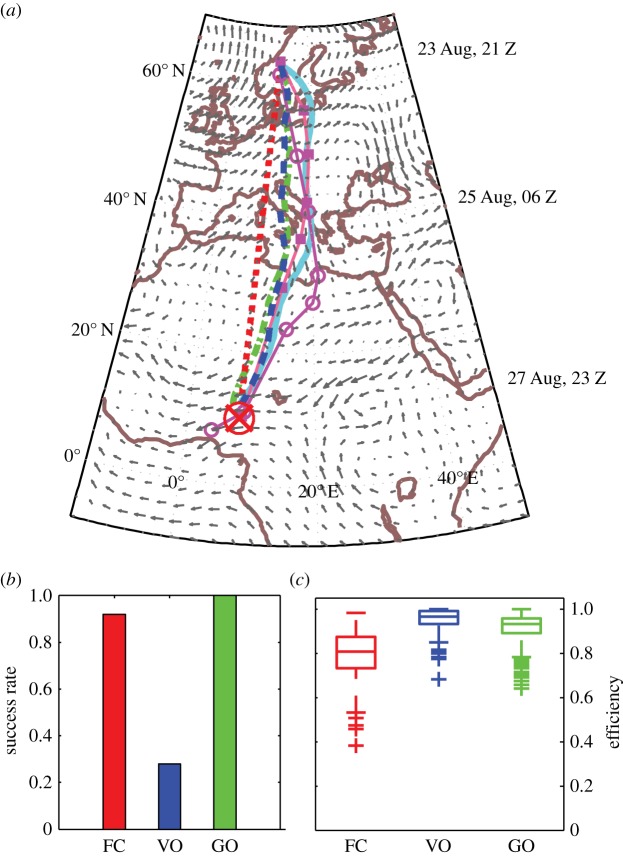
Simulated non-stop great snipe migration to Africa. Simulated trajectories and trajectories inferred from geolocator studies of great snipes departing Scandinavia on 30 August 2010 (*a*) to within a 250 km radius (outlined in red) of a goal located in West Africa (red cross), and considering 33 seasons of simulations (16–30 August 1979–2011), success rates (*b*) and boxplots of efficiency (*c*) for each strategy: optimal orientation (cyan lines), vector orientation (VO, dashed blue lines), goal orientation (GO, dot-dashed green lines) and full compensation (FC, dotted red lines). Great snipe trajectories inferred from geolocator data are indicated with pink lines, with flight durations of 72 h (filled squares) and 84 h (open circles). Wind quivers (grey arrows) depict wind speed and direction are scaled to 26 m s^−1^ and synchronized with optimally orienting migrants at the same latitude (see time stamps on right of maps).

Results for great snipe simulations are summarized in figure 5, with simulated and tracked great snipe trajectories departing 23 August 2009 (*W* = 0.6, figure 5*a*) and, for 33 seasons of simulated flights, success rate (figure 5*b*) and boxplots of efficiency (figure 5*c*) for each strategy. With the exception of full compensation, simulated trajectories, flight durations (and efficiencies) for this departure date were similar: 77 h (1.0) with optimal orientation, 80 h (0.96) with vector orientation and 84 h (0.92) with goal orientation. Full compensation took considerably more time 107 h (*ɛ* = 0.72). Two great snipes tracked with geolocators on this date (pink tracks with symbols in figure 5*a*) were even faster (more efficient) than optimal simulations (*ɛ* = 1.04 and 1.10). This is presumably attributable to higher airspeeds and/or superior altitude selectivity by the tagged individuals. The estimated track of the faster tagged individual (with filled square markers) was nonetheless tantalizing similar to that with optimal orientation. Considering all 33 seasons of simulated flights, vector orientation (with constant flow-adjusted heading of 190.6°) was slightly but significantly more efficient (median efficiency 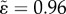) than both full compensation (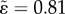, Wilcoxon's signed rank 4, *p* < 10^−23^) and goal orientation (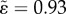, Wilcoxon's signed rank 1435, *p* < 10^−10^), but also the least reliable in arriving within 250 km of the goal (*P_a_* = 0.28 versus 0.92 and 1.0 with full compensation and goal orientation, respectively). Simulated vector-orienting great snipes did however typically pass relatively close to the goal, with a median (and quartile range) in closest approach among departure dates of 500 km (220–800 km, i.e. 3–14% of the initial goal distance).

## 4. Discussion

In this study, we have introduced optimal orientation as a benchmark for evaluating the performance of orientation strategies in given flow conditions. With optimal orientation, trajectories are longest yet travel duration is minimized by steering through regions of relatively high flow support. The illustrated trajectories (figures 1*a*,*b* and 2*a*,*b*) demonstrate that optimal orientation in horizontal flow does not always involve increased compensation on approach to the goal (cf. [12,13]), can involve either over-drift or over-compensation en route (cf. [23,38]) and is not always equivalent to full drift in ‘balanced’ flows (contra [12]).

Our results provide insight into the value of information an animal may have about flow conditions. In flow that is weak compared to self-speeds (*W* ≤ 0.5), flow prediction is not essential as full compensation and goal orientation are all nearly as reliable and efficient as flow-adjusted vector orientation and optimal orientation (figures 1*d*, 2*d* and 3*d*). This finding is consistent with evidence of selectivity for low flow speeds among migratory taxa [6,50–52]. In stronger flow, animals have much more to gain from explicit or intrinsic information about flow patterns, as evidenced by the sometimes dramatic differences in performance between on the one hand optimal orientation and flow-adjusted vector orientation, and on the other hand full compensation and goal orientation. Vector orientation involving pro-active adjustment of headings was shown to be more efficient than either goal orientation (i.e. strictly following a map sense) or full compensation in strong and variable flow, and performed nearly as well as optimal orientation. Naturally, if flow cannot be predicted *a priori*, adjusting headings en route can be advantageous in avoiding unnecessary barriers [36,53], reorienting following unanticipated drift [54,55] or to avoid becoming trapped in strong rotational flow (see figure 3*b* and cf. [42,56]).

The comparison between two natural migration systems reveals interesting contrasts in the effect of spatio-temporal scales on the performance of orientation strategies. With the relatively short thrush flights, during which we could expect wind conditions to be somewhat predictable, vector orientation is nearly time-optimal and clearly outperforms full compensation and goal orientation strategies, especially under strong flow conditions. Interestingly, at the much larger spatio-temporal scales of the great snipe flights, during which flow conditions throughout the flight are unlikely to be predictable, goal orientation is nearly optimally efficient. This probably stems from two factors: (i) the high airspeeds of great snipes (*ca* 20 m s^−1^ [45]) diminishing the effect of lateral winds and (ii) reduced spatial coherency of experienced flow, limiting the potential advantage of flow predictability. This further suggests that to the extent that great snipe have continual access to navigational cues, they can travel over large distances reliably and nearly optimally fast by heading towards the goal. The routes of the snipes tracked with geolocators apparently curved to the east similar to optimally orientating trajectories, although more detailed and accurate tracks, including information about headings, would be required to unravel the orientation and navigation behaviour of these birds during their astonishing flights.

Therefore, the extent to which taxa can approach optimal orientation will depend both on the scale and strength of flow relative to motion and navigation capacities [3,57] and on abilities to gauge and predict flow. Although assessing limitations of and transitions between navigational cues goes beyond the scope of this study, it is relevant to note that long-distance migration is generally thought to require different navigational cues [34] at various spatial scales to ensure arrival [35]. In this context, the simplicity, near-optimal efficiency and relatively close approach to the goal (median 500 km) of the great snipe simulations based on endogenous headings support the notion that vector orientation can provide a basis for long-distance movements, as proposed for Nearctic–Neotropical landbird migration over the Atlantic Ocean [37] and monarch butterfly *Danaus plexippus* migration [58]. Regarding abilities to gauge and predict flow, swimming animals may be particularly constrained [6], and ocean currents may in fact be even less predictable than in the atmosphere (travel distances being similar [3] but synoptic scales shorter [59,60]). Consistent with such flow unpredictability, migration routes to foraging grounds by loggerhead turtles (*Caretta caretta*) were recently found to match goal-orienting more closely than time-optimal routes [61].

Assessing optimal orientation in flows remains computationally challenging and further limited by availability and accuracy of flow data (cf. [62]) and by uncertainties regarding effects of vertical movements, variable self-speed and energy considerations. Results from this study suggest that in weak flows (e.g. overall less than half the self-speed), the optimal benchmark could be approximated by calculating flow-adjusted vector orientation. Selection of vertical layers is obviously also relevant [19,23,24], but for example among migrating birds, altitudes with the highest instantaneous flow support are not always selected [20]. Adjustment of self-speed to horizontal flow can be of considerable energetic importance [12,51], but the degree of such adjustment remains obscure [63,64]. The adaptive benefit of adjusting self-speed will depend on trade-offs between time and energy expenditure [65], but may furthermore be physiologically constrained [66,67] or superseded by the selection of favourable flow conditions [36,68].

The myriad of animal movement data made available through modern tracking technology offers great potential for understanding the role of flow-orientation in animal movement but also raises a challenge to interpretation [63,69]. The highly contrasting patterns of compensation that can arise in various flow patterns (figures 1–3) emphasize the importance of not only assessing reaction to flow and flow support instantaneously in relation to track directions or implied preferred directions [4,64,70], but also over the entire scale of movement. Comparing optimal and observed orientation over entire routes allows estimation of the potential and realized flow support and potential insight into the navigational capacities and flow-predictive abilities among swimming and flying migrants.

## Figures and Tables

**Table:** 

Advection	Motion induced by the ambient flow.
Balanced flow	Flow for which full drift results in no cumulative lateral drift.
Direction of travel	The angle of an animal's trajectory (clockwise from geographic north).
Compensation	Adjustment of self-speed and/or heading to prevent or diminish lateral drift. Simulations in this study presume constant self-speeds.
Drift	Advection by lateral flow, i.e. perpendicular to the migrant's preferred direction. The term passive drift, sometimes used to describe movement in water, refers to drift without self-speed.
Endogenous heading	An inherited preferred migratory direction or heading (in the latter case possibly adapted to prevalent flow).
Flow support	Travel speed minus self-speed, i.e. the difference in speed from that in the absence of flow. Hence we quantify flow support over the entire trajectory as opposed to locally (cf. local measures of flow assistance in [4]).
Flow strength	Ratio of flow speed to self-speed.
Full compensation	Reaction to flow, restricted in this study through adjustment of heading, resulting in a constant direction of travel, i.e. precluding drift. Only possible when the self-speed exceeds the lateral flow speed component relative to the heading.
Full drift	Non-adjustment to flow, i.e. continually heading in the initial goal direction.
Goal orientation	An orientation strategy whereby the animal repeatedly heads towards the goal. This does not preclude drift, i.e. goal orientation is distinct from full compensation.
Ground speed	An animal's instantaneous (horizontal) speed relative to the ground.
Heading	The angle of an animal's body axis relative to the ground (clockwise from geographic north).
Lateral flow	The flow component perpendicular to a particular direction, here the preferred, i.e. goal direction.
Navigation	The process of reaching a remote goal from familiar or unfamiliar locations.
Optimal orientation	Time-minimizing orientation in any horizontal flow, resulting in fastest arrival at the goal.
Orientation	The determination and maintenance of heading relative to the ground.
Orientation strategy	An orientation behaviour to negotiate flow, typically involving endogenous headings (see vector orientation) and/or exogenous factors (in this study, flow conditions and navigational cues).
Over-compensation	Adjustment of heading resulting in lateral drift opposite to that of the ambient flow.
Over-drift	Adjustment of heading which increases lateral drift.
Preferred direction	The intended travel direction. In this study, the preferred direction is ultimately the goal direction but headings differ instantaneously according to the orientation strategy and flow configuration.
Self-speed	An animal's self-propelled speed relative to the moving flow (not adjusted to flow in this study). Typically referred to as the airspeed and swim speed in air and water, respectively.
Shear flow	A gradient in flow speed along any direction; here used to mean a gradient in lateral flow along the initial goal direction.
Travel speed	Speed of travel including non-movement periods. With non-stop movement, equivalent to mean ground speed.
True navigation	The ability to determine the direction to the goal from anywhere within the navigable range, even when drifted or displaced from intended paths.
Vector orientation	An orientation strategy, characterized by (a sequence of) constant headings. Typically proposed to be a juvenile migration strategy, we here also simulate vector orientation by animals navigating in predictable flow by allowing headings to be adjusted on departure according to goal location and flow conditions en route.

## Appendix A. Generic orientation strategies

Full compensation involves heading into the flow to such an extent that the track and goal directions are aligned, thus instantly negating any lateral drift [4]. Foraging fish in swift rivers have for example been shown to fully compensate over short-distances [71]. However, full compensation is only possible if flow speeds do not exceed self-speeds (in this case, simulations were terminated). Vector orientation involves choosing a single heading on departure, based on actual (or prevailing) flow conditions, to ensure (or maximize the likelihood of) successful arrival. This is an extension of vector orientation as typically proposed, i.e. as an orientation strategy of juvenile migrants with no or limited map sense [53] and being insufficient to reach specific goals [72,73]. However, by choosing a heading that compensates for the cumulative drift over the resulting route an animal could reach a goal destination. For example, Neotropical migrants have been proposed to take advantage of easterly trade winds to reach South America via eastern North America on constant headings [37]. A special case of vector orientation is in balanced flow, i.e. where simply heading in the initial goal direction results in no cumulative lateral drift, in which case it is equivalent to full drift. The third strategy, goal orientation, involves heading continually towards the goal regardless of displacement by drift. This is equivalent to a full drift strategy with continually updated preferred direction [4] but is also essentially equivalent to a strategy that minimizes the goal distance in infinitesimal steps (cf. [13]). Over entire routes, goal orientation has been shown to be suboptimal in strong uniform flow (e.g. [13]), but it may be relevant when gauging drift is unreliable or impossible [6,32].
